# Effects of age on H1N1-specific serum IgG1 and IgG3 levels evaluated during the 2011–2012 influenza vaccine season

**DOI:** 10.1186/1742-4933-10-14

**Published:** 2013-04-22

**Authors:** Daniela Frasca, Alain Diaz, Maria Romero, Nicholas V Mendez, Ana Marie Landin, Bonnie B Blomberg

**Affiliations:** 1 Department of Microbiology and Immunology, University of Miami Miller School of Medicine, P.O. Box 016960 (R-138), Miami, FL 33101, USA

**Keywords:** Aging, B cells, Influenza vaccine response

## Abstract

**Background:**

We have previously reported an age-related impairment in the serum antibody response to pandemic (p)2009 H1N1, measured by hemagglutination inhibition assay and ELISA. The present study extends these observations and evaluates IgG subclass distribution in healthy individuals of different ages vaccinated during the 2011–2012 season.

**Results:**

The 2011-2012 vaccination season was characterized by a vaccine containing the pandemic (p)2009 H1N1 strain for the third consecutive year. All of our subjects were previously immunized, and therefore seroprotected at t0. Nevertheless, aging impaired the serum antibody response to H1N1, as antibody titers increased after vaccination in young and less in elderly individuals. The peak of the response was at day 7 (t7), in contrast with what is usually seen at day 21–28, suggesting a memory response characterized by the induction of an IgG subclass with a shorter half-life. We hypothesized that the IgG3 response, with its much shorter half-life, might be more represented. Antibodies were predominantly of the IgG1 subclass in both age groups, although a robust IgG3 response was also induced and accounted for a significant proportion of the overall response. IgG2 and IgG4 antibodies were at indiscernible levels. We showed a much higher percentage of IgG3 (40–50%) than previously in the literature (less than 10%). To explain if this was associated with a particular cytokine profile, we measured H1N1-induced T cell cytokines in vitro and found that IgG3 levels were positively correlated with TNF-α and IL-6. Moreover, activation-induced cytidine deaminase (AID) mRNA expression, a predictive biomarker of optimal in vivo vaccine response, was found to significantly correlate with IgG3 and also with IgG1 similar to what we have shown previously for total IgG.

**Conclusions:**

In the 2011–2012 season, the pandemic (p)2009 H1N1 strain was present in the vaccine for the third consecutive year and therefore each individual was seroprotected at t0. The peak of the response was at t7, suggesting a memory response characterized by a robust induction of IgG3, which was associated with TNF-α and IL-6 production. Both IgG1 and IgG3 responses were decreased by age. AID was confirmed to be a predictive biomarker of optimal vaccine responses.

## Background

Influenza is associated with morbidity and mortality in individuals 65 years of age and older, children under 2 years of age and individuals at high risk for complications from influenza because of other co-morbidities [1]. These complications may include secondary bacterial infections, exacerbations of pre-existing medical conditions [1,2] and hospitalization which significantly contributes to the development of disability in the elderly [3] and represent a significant economic burden [4]. Influenza infections every year are associated with approximately 200,000 estimated hospitalizations and 36,000 deaths in the United States [5].

The effects of influenza vaccination are different in individuals of different ages [6-8]. Successive annual vaccinations increase protection against influenza [9,10]. There is evidence that elderly individuals who have routinely received the vaccine can still contract the infection, with secondary complications leading to hospitalization, physical debilitation, exacerbation of underlying medical conditions and death [11-13]. However, influenza vaccines reduce the severity of the infection and also prevent complications from influenza and therefore vaccination campaigns targeted to improve immune functions in elderly individuals are supported.

Immunoglobulin (Ig) G levels are important for antibody vaccine responses and the subclass distribution may be clinically relevant, as suggested by the evidence that IgG subclass deficiencies are associated with severe influenza infections in immunocompromised patients [14]. In humans there are four subclasses of IgG: IgG1, IgG2, IgG3 and IgG4, which constitute about 65%, 23%, 8%, and 4%, respectively, of the total serum IgG. The four subclasses differ in structure, half-life and function. IgG1 and IgG3 are the most important in complement-fixation and antibody-dependent cellular cytotoxicity, which have a role in virus neutralization. Previous studies have shown that the major subclass of IgG detected in serum after influenza infection or vaccination is IgG1 and then IgG2 [15] or IgG3 [16-20] , but in much lower amounts. Levels of IgG4 are usually lower than those of the other subclasses.

Both vaccine and host factors influence the serum IgG subclass distribution after influenza vaccination. Soluble peptides preferentially induce IgGl and IgG3 responses [21,22], whereas natural infection or vaccination with live and/or inactivated viruses induce predominantly if not exclusively IgG1 [18,19]. Endogenous cytokines may also play an important role in regulating Ig isotype production. In general, IgG1 is associated with a Th2 profile and the other subclasses are mainly associated with a Th1 profile [23].

Aging has been associated with a significant impairment of IgG1, but not IgG3 production [20] and this has been suggested to be one of the causes of the lower influenza vaccine efficacy in the elderly perhaps due to the lower half-life of IgG3 antibodies. Other reports showed no IgG3 response to the vaccine in either young or elderly [15,24]. Changes in cytokine profile during senescence have been reported and attributed to changes in both the innate and the adaptive immune systems [25-28], which may cause parallel changes in the relative amounts of the different IgG subclasses produced in response to the influenza vaccine.

In the present study we have investigated the effects of age on serum IgG subclass responses after vaccination of individuals of different ages with the trivalent inactivated influenza (TIV) vaccine, during the 2011-2012 season. This vaccination season was characterized by a vaccine containing the pandemic (p)2009 H1N1 strain for the third consecutive year. We measured the H1N1-specific response and noticed that the peak of the response was at t7 which we hypothesized could be due to both increased IgG3 serum levels as well as to the restimulation of a memory response. IgG1 was the main IgG subclass detected in serum after influenza vaccination, followed by IgG3, confirming previously published data [16-20], but there was much more, up to 50%, IgG3 in our studies. Moreover, aging decreased both IgG1 and IgG3 serum levels, as opposed to what has been previously shown [20]. In order to evaluate the cytokine profile induced by the vaccine and associated with these elevated IgG3 serum levels, we stimulated PBMC with H1N1 and found significant correlations between IgG3 levels and TNF-α and IL-6 production. We also measured activation-induced cytidine deaminase (AID), a biomarker of optimal antibody responses, and made correlations with IgG1 and IgG3 serum levels. AID is essential for DNA cleavage required for class switch recombination (CSR) and somatic hypermutation (SHM) which are crucial for the generation of high affinity antibodies and robust humoral immunity [29,30]. CSR and SHM occur in germinal center B cells in response to both T-dependent and T-independent stimuli [31,32]. AID triggers CSR and SHM by deaminating cytosines in the variable and switch regions of the Ig locus, converting them to uracils, and the resulting mismatches are recognized by specific enzymes and excised, leading to DNA double strand breaks [33]. Our results herein show that AID is positively correlated with IgG3 antibodies, and also with IgG1, similar to what we have shown previously for total IgG.

## Results and discussion

### Characteristics and serological profile of the subjects in the study

Demographic and basic information on the participants (age, gender, race, ethnicity) and their serological profiles are in Table 1. We measured serum levels of TNF-α, IL-6, CRP as well as cytomegalovirus (CMV) positivity. Results show significantly higher levels of serum TNF-α, IL-6 and CRP in elderly as compared to young individuals. The percentage of CMV-positive individuals was also significantly higher in the elderly group.

**Table 1:** Demographic and serological characteristics of the participants

	**Young**	**Elderly**
Participants (n)	35	25
Age (mean years ± SE)	40 ± 3	69 ± 5^b^
Gender (M/F)	12/23	11/14
Race (W/B)	20/15	15/10
Ethnic Categories (Hispanic/Non Hispanic)	14/21	8/17
CMV (% positive individuals)	22	78^b^
TNF-α (pg/ml)	6 ± 1	13 ± 4^a^
IL-6 (pg/ml)	44 ± 18	101 ± 33^b^
CRP (pg/ml)	640 ± 95	895 ± 57^b^

Young: 20-59 years, Elderly: ≥60 years.

^a^p < 0.01, ^b^p < 0.05.

### Age effects on in vivo vaccine responses

**Figure 1:**
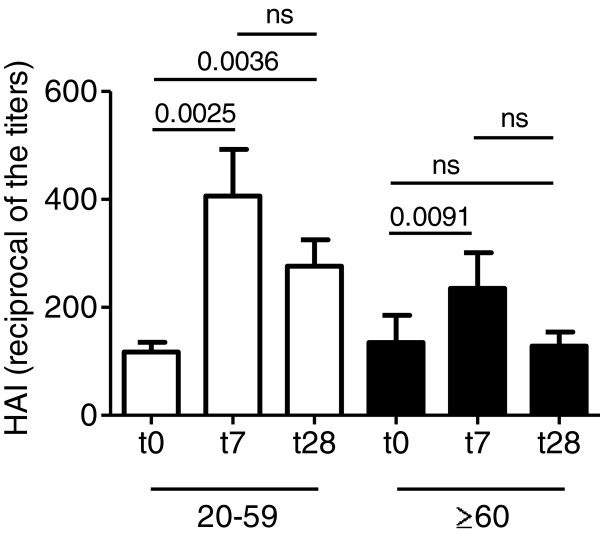
**Decreased serum response to the influenza vaccine in the elderly.** Sera isolated before (t0) and after vaccination (t7 and t28) were evaluated in HAI. Results are expressed as reciprocal of the titers. Non-parametric analyses were calculated by Mann-Whitney test (two-tailed), using GraphPad Prism 5 software. See text for differences between young and old groups. The numbers of individuals are given in Table 1.

We measured the in vivo response to the vaccine using the HAI assay which is the best correlate for vaccine protection. In addition, we also measured the serum response by ELISA and we have previously shown that it completely correlates with HAI [34]. We tested the purified proteins from the 3 viral strains present in the vaccine preparation: H1N1, H3N2 and B. Here, we only present data on H1N1 because the response to H3N2 and B was much lower and we wanted to measure the memory response to the H1N1 antigen given the third time. The response to the antigen H3N2 followed the same pattern as that of H1N1, whereas the response to the antigen B did not show age-related differences between young and elderly individuals (see below). Results in Figure 1 show similar titers in young and elderly individuals at t0, due to the fact that all individuals were vaccinated in the previous 2 seasons with a vaccine containing the same H1N1 p2009 swine-origin strain (p = 0.41). Titers increased significantly in young and to a lesser extent in elderly individuals at t7, but at t28 increased significantly only in young individuals. At t7 and t28, titers in young and in elderly individuals were significantly different (p = 0.03 and p = 0.001, respectively). Nevertheless, all subjects had titers at t0 ≥1:40, suggesting protection from potential influenza infections. This result is different from what we and others have previously shown, where the optimal response to vaccination in both young and elderly individuals was at t28 and was sustained until 12 weeks post-vaccination [35,36].

In young individuals, the H3N2-specific response increased at t7 and t28 as compared to t0. Titers were: 210 ± 42 (t0), 833 ± 165 (t7) and 668 ± 165 (t28). Differences between t0 and t7/t28 were statistically significant (p < 0.05 both). In elderly individuals, the response to H3N2 did not increase at t7 or at t28 as compared to t0. Titers were: 232 ± 49 (t0), 374 ± 101 (t7) and 357 ± 64 (t28). Therefore, at the peak of the response (t7) the H3N2-specific titers were significantly decreased by aging (p < 0.05). The B-specific response was comparable in young versus elderly individuals at t0 (210 ± 55 versus 275 ± 66) and did not increase after vaccination at t7 (273 ± 48 versus 187 ± 42) or t28 (301 ± 84 versus 297 ± 59).

**Figure 2:**
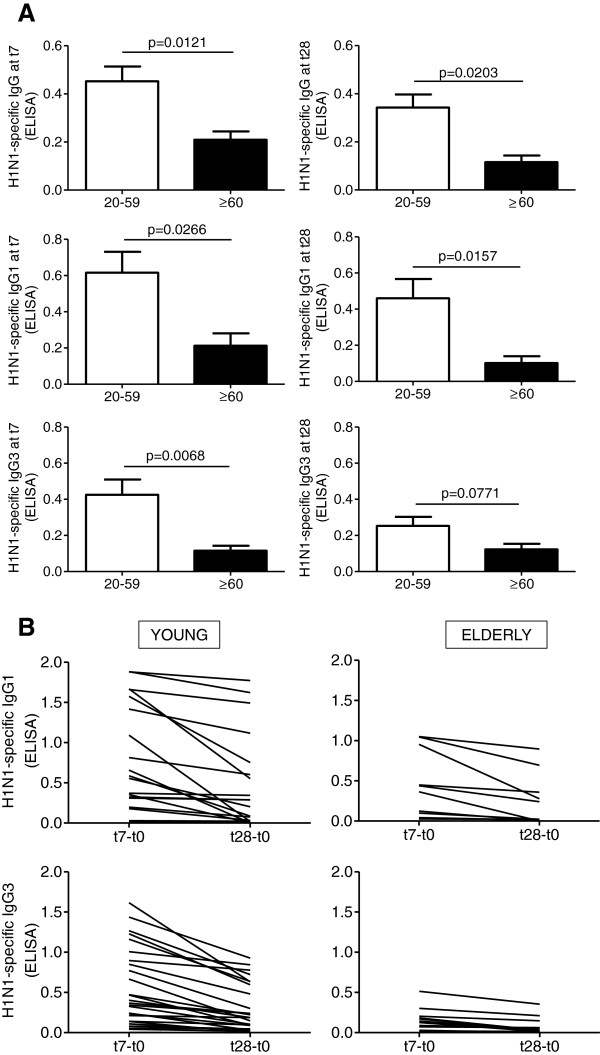
**Decreased induction of IgG, IgG1 and IgG3 by the influenza vaccine in the elderly. A.** Sera isolated before (t0) and after vaccination (t7 or t28) were collected and evaluated in H1N1-specific IgG, IgG1 and IgG3 ELISA. Data are expressed as OD at t7 or t28 *minus* OD at t0. All values here demonstrate a positive response. Data would look similar if only OD at t7 or t28 are shown. Mean comparisons between groups were performed by Mann-Whitney test (two-tailed), using GraphPad Prism 5 software. P values (two-tailed) are indicated in each graph. The numbers of individuals are given in Table 1. **B.** Individual IgG1 and IgG3 responses at t7 and t28 are shown. The numbers of individuals are given in Table 1.

The fact that in both young and elderly individuals the antibody titers didn’t increase or stay sustained after t7 suggested stimulation of an existing memory response to this vaccine and also that an IgG subclass with a shorter half-life may have been induced. We therefore measured total IgG and the 4 different IgG subclasses at t7 and t28 by H1N1-specific ELISA. Only antibodies of IgG1 and IgG3 subclasses were detected in serum. Results in Figure 2A show that aging significantly impairs IgG serum levels at t7 and t28, and we have already shown this for t28 [34] and responses are lower at t28 for young (p = 0.045) as well as for elderly individuals (p = 0.041). We measured the subclass responses here by ELISA because we cannot measure the subclasses by HAI and we have previously shown that the HAI and ELISA responses completely correlate [34]. Aging also significantly decreases IgG1 serum levels at t7 and t28 and IgG3 serum levels at t7 and responses are lower at t28 for young (p = 0.035) and also for elderly individuals (p = 0.042). We found that the peak response was at t7 for both subclasses, and also for IgG, and we expected this kinetic for IgG3 but not for IgG1, due to the different half-lives of the 2 subclasses (7 and 21 days, respectively). Antibodies induced by vaccination were predominantly of the IgG1 subclass in both age groups, although a robust IgG3 response was also induced and accounted for a significant proportion of the overall serologic response to the influenza vaccine in both age groups. The percentage of individuals with a predominant IgG1 response was 70% and 62% in young and elderly, respectively, whereas that of individuals with a predominant IgG3 response was 12% and 25%, in young and elderly, respectively. The percentage of individuals with equal amounts of IgG1 and IgG3 was 18% and 13% in young and elderly, respectively. The decrease in serum levels of IgG1 and IgG3 subclasses at t28 as compared to t7 is shown in Figure 2B for each individual and this decrease is significant only for young (p = 0.025) but not for elderly individuals (p = 0.071). These results suggest that the reason why the serum response did not increase or remained sustained at t28 cannot exclusively depend on the induction of an IgG subclass with a shorter half-life (IgG3).

We hypothesize that the vaccine composition in the 2010-2011 and 2011-2012 seasons induced a higher IgG3 serum response, as samples that we have tested in previous seasons, including the p2009 season, induced almost no IgG3. In particular, pH1N1-specific IgG3 were detectable only at t7. ELISA values, expressed as OD at t7 *minus* OD at t0, were: 0.11 ± 0.01 and 0.08 ± 0.01 in young versus old. Because the total IgG serum response was higher due to repeated immunizations (see Figure 1), the absolute amount as well as the percentage of the response was increased for IgG3. These studies suggest that both the humoral and cellular immune response to particular vaccines should be more completely followed and correlated with protection. The high IgG3 levels in the 2011-2012 season were not deleterious, as the IgG1 levels were also high and the vaccine likely generated a good T cell response. In fact, according to Centers for Disease Control and Prevention (CDC) the 2011-2012 season set a new record for the lowest peak for influenza-like-illness ever recorded [37].

We also measured during the 2011-2012 season the IgG serum response to the other 2 antigens present in the vaccine: H3N2 and B by ELISA. Results indicate that at the peak of the response (t7) the H3N2-specific IgG response was significantly decreased by aging (0.321 ± 0.046 and 0.125 ± 0.013, in young versus elderly, respectively, p < 0.05) whereas the B-specific IgG response was comparable in both age groups (0.122 ± 0.006 and 0.125 ± 0.012, in young versus elderly). These responses were lower than the H1N1 response and therefore we didn’t measure the subclasses specific for these antigens.

### Age effects on in vitro cell mediated immunity

**Figure 3:**
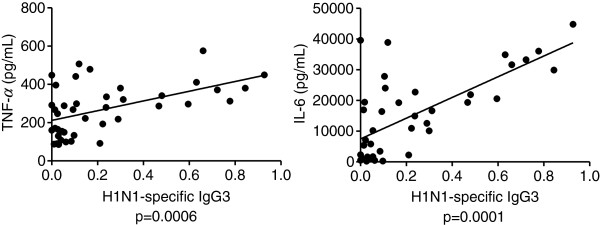
**Pro-inflammatory cytokine production in cultured PBMC is positively correlated with levels of serum H1N1-specific IgG3.** Frozen PBMC (10^6^ cells/ml), isolated from the peripheral blood at t0, were thawed and cultured with H1N1 (2 μg), for 3 days. At the end of this time, supernatants were collected and analyzed in ELISA for cytokine production. Correlations were calculated by Pearson’s test, using GraphPad Prism 5 software. P values are indicated for each graph. The numbers of individuals are given in Table 1.

To understand if the IgG1/IgG3 production was associated with a particular cytokine profile, we then measured H1N1-induced T cell cytokine production in H1N1-stimulated PBMC cultures. H1N1 was the only antigen able to stimulate T cell cytokine secretion in cultures of PBMC. We measured IFN-γ, TNF-α, IL-4, IL-5, IL-6, IL-10 and Blys and we correlated these with the IgG1 and IgG3 serum levels at t7 and t28. We found significant positive correlations only between TNF-α and IL-6 and IgG3 levels at t28 (Figure 3). A positive trend was observed between IL-10 and IgG3 levels, but it was borderline of significance (p = 0.058). Conversely, Blys and IgG3 levels showed a negative trend, which was not statistically significant (p = 0.38). All correlations and p values are in Table 2. These results indicate that IgG3 production is associated with a pro-inflammatory cytokine profile. For T cells, production of pro-inflammatory cytokines in response to a vaccine is beneficial. Therefore, the suboptimal response with IgG3 here could be compensated by a better T cell response to the vaccine and would be protective for the individual.

**Table 2:** Correlations between cytokine production in H1N1-stimulated PBMC cultures and serum IgG3 levels

**Cytokine in supernatants**	**IgG3 (t7) Person’s r**	**p**	**IgG3 (t28) Person’s r**	**p**
**TNF-α**	0.0861	0.58	0.3545	**0.0006**
**IL-6**	0.2264	0.31	0.4556	**0.0001**
IL-10	0.3120	0.09	0.3963	0.06
IFN-γ	0.1623	0.96	0.1918	0.91
IL-4	0.0215	0.56	0.0467	0.49
IL-5	0.0484	0.43	0.0513	0.40
Blys	−0.1604	0.16	−0.1528	0.38

PBMC (10^6^ cells/ml) were isolated from the peripheral blood at t0, thawed and cultured with H1N1 (2 μg), for 3 days. At the end of this time, supernatants were collected and analyzed in ELISA for cytokine production. Correlations were calculated by Pearson’s test, using GraphPad Prism 5 software.

Immunosenescence is associated with a low grade chronic pro-inflammatory status called inflammaging [27]. We can hypothesize that increased levels of pro-inflammatory mediators with age will induce predominantly the release of IgG3 antibodies which last for a shorter time as compared to IgG1 antibodies and therefore will be less protective than IgG1. However, this was not the case during the 2011-2012 flu season, due to the fact that H1N1 was not a new antigen and every individual had a protective titer at t0. In future vaccination seasons in which a new antigen will be part of the vaccine, it will be necessary to evaluate whether inflammaging will induce the release of robust IgG3 production and whether these antibodies will be effective in protecting individuals at risk from infection with influenza viruses.

### AID is also positively correlated with IgG3

We have recently identified B cell-specific biomarkers which can be used to predict the in vivo response to the influenza vaccine. The enzyme AID is one of our chosen markers because we have previously shown that it completely correlates with the ability of human B cells to undergo CSR [38] and affinity maturation of Ig genes [39].

**Figure 4:**
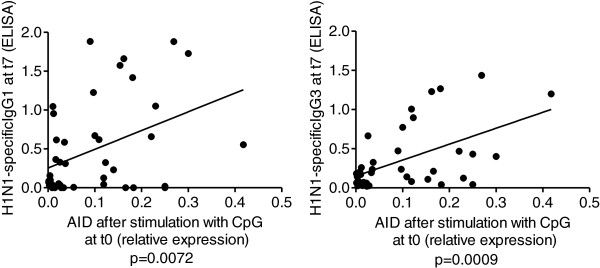
**CpG-induced AID at t0 and the serum response are correlated.** Frozen PBMC (10^6^ cells/ml), isolated from the peripheral blood at t0, were thawed and cultured with CpG, for 3 days. At the end of this time, cells were processed as described in Methods. Results are expressed as raw qPCR values (ΔCt) of AID mRNA normalized to GAPDH. Correlations between CpG-induced AID at t0 and H1N1-specific ELISA were calculated using GraphPad Prism 5 software. Pearson’s r = 0.4086 (left) and r = 0.5249 (right). P values are indicated for each graph. The numbers of individuals are given in Table 1.

We measured AID mRNA expression induced by CpG in the same individuals as above. The CpG response was measured at t0, as we have shown that this can predict the robustness of the in vivo vaccine response [34]. Results in Figure 4 show that AID mRNA expression in response to CpG at t0 significantly correlates with the in vivo response measured by H1N1-specific serum IgG3 as well as IgG1 levels at t7 and this also occurs at t28 (for IgG3, Pearson’s r = 0.3921, p = 0.035; for IgG1, Pearson’s r = 0.4025, p = 0.021). These results confirm our previous evidence that CpG-induced AID measures the competence of B cells and can effectively predict the ability of an individual to generate optimal specific humoral responses, which reflect not only expansion of previously induced switched memory B cells and ultimately generation of long-lived plasma cells, but also an ongoing switch process from IgM memory to switched memory B cells and we have shown herein that IgG3 switch is associated with production of pro-inflammatory cytokines.

## Conclusions

Because of repeated immunizations, the total IgG response in the 2011-2012 season to the pandemic (p)2009 H1N1 strain was an excellent, protective response for every individual. In fact, the CDC has stated that this was the year with the lowest incidence of influenza-like-illnesses. In analyzing this response, our data showed that the peak of the response was at day 7, suggesting a memory response. This response was characterized by a robust induction of IgG3, which is known to have a shorter half-life than IgG1. Presently, it is not clear what contributed to this increased IgG3 response, but presumably components in the vaccine preparation itself. It is important to know that both IgG1 and IgG3 were increased after vaccination in all individuals, and thus would have contributed to their protection. In an attempt to explain why there was more IgG3, we measured in vitro generated T cell cytokines and found that the H1N1-specific serum IgG3 levels were associated with TNF-α and IL-6. Both IgG1 and IgG3 responses were decreased by age and we confirmed that AID generated in vitro after stimulation is a predictive biomarker of not only IgG1 but also IgG3 in vivo vaccine responses.

## Methods

### Subjects

Experiments were conducted using blood isolated from healthy volunteers of different ages after appropriate signed informed consent and were approved with IRB protocol #20070481. In every experiment reported in the present paper, 35 young and 25 elderly individuals were evaluated. For the purpose of this study, “elderly” individuals refer to individuals ≥60 years of age. We previously reported that individuals of this age may be categorized in the “elderly/affected” group [34,35,38,39], as B cell numbers and functions (antibody production and affinity maturation) are significantly decreased. Demographic and serological characteristics of the participants are shown in Table 1. All subjects were influenza-free at the time of enrollment, at the time of blood draws, were without symptoms associated with respiratory infections, and did not contract flu-like symptoms within a 6 month follow-up period.

### Influenza vaccination

The study was conducted during the 2011-2012 seasonal influenza vaccination. Two Trivalent Inactivated Vaccines (TIV) were used: Novartis Fluvirin and GSK Fluarix. Blood samples were collected immediately before vaccination (t0), one week (t7) and 4-6 (t28) weeks post-vaccination. The in vivo response to the influenza vaccine was measured by HAI (Hemagglutination inhibition) assay and by ELISA.

### Hemagglutination inhibition (HAI) assay

We evaluated the response to the influenza vaccine by fold-increase in the reciprocal of H1N1-specific titers at t7 divided by that at t0 by HAI assay, as we have previously described [34,35]. The H1N1 2009 egg-derived mono-bulk subunit antigen (A/California/07/2009) from Novartis Vaccines and Diagnostic (Siena, Italy) was used at the concentration of 2 μg/ml. The H3N2 and B antigens, also from Novartis, were used at the same concentration. The HAI assay is based on the ability of certain viruses or viral components to hemagglutinate the red blood cells of specific animal species [40]. Antibodies specific to influenza can inhibit this agglutination. Paired pre- and post-immunization serum samples from the same individual were tested simultaneously. Briefly, sera were pretreated with receptor destroying enzyme (RDE, Denke Seiken Co Ltd) for 20 hrs at 37°C; in order to inactivate this enzyme, sera were then heated at 56°C for 60 min. Two-fold serial dilutions were done; 25 μl of diluted sera were incubated with an equal volume of 4 HA units of the H1N1 antigen, for 1 hr at room temperature and then 50 μl of a 1.25% suspension of chicken red blood cells were added. After two hrs of incubation at room temperature titers were determined. Serum inhibiting titers of 1/40 or greater are the defined positive measure of seroprotection against infection, whereas a four-fold rise in the reciprocal of the titer from t0 to t28 indicates a positive response to the vaccine and indicates seroconversion [34,35,40,41]. Because the 2011-2012 season was the third consecutive one in which the H1N1 was the pandemic 2009 swine-origin strain, we saw an early serum response to the vaccine which peaked 7 days after vaccination. For the same reason, initial titers were ≥1/40 in 94% of the individuals in the study, indicating high levels of seroprotection.

### Enzyme-linked immunosorbent assay (ELISA)

H1N1-specific IgG concentrations in serum of individuals before and after vaccination were evaluated by human Ig quantitative ELISA kits (Bethyl Labs E80-104). H1N1-specific IgG1 and IgG3 were measured by human Ig quantitative ELISA kits (Southern Biotech, 9052-08 and 9210-04, respectively), after coating the plates with the H1N1 subunit antigen (Novartis), at the concentration of 2 μg/ml. Results are expressed as follows: OD values at t7 (or at t28) - OD values at t0.

TNF-α, IL-6 and CRP were also measured by ELISA kits (Life Technologies KHC3013, KHC0062 and KHA0032).

CMV serostatus was determined by ELISA using the CMV-IgG-ELISA PKS assay (Medac), which measures serum CMV-specific IgG antibody levels.

IFN-γ, TNF-α, IL-4, IL-5, IL-6 and IL-10 were measured by human ELISA kits (Life Technologies KHC4121, KHC3013, KHC0043, KHC0052, KHC0063, KHC0103, respectively). Blys was measured by the hypersensitive ELISA kit (adipoGen AG-45B-0001-KI01).

### PBMC cultures

PBMC were collected by density gradient centrifugation using Vacutainer CPT tubes (BD 362761). Cells were then washed and cryopreserved (frozen) with 90% fetal bovine serum and 10% DMSO. Frozen PBMC (10^6^) were processed and stimulated for 1-3 days with H1N1 at the concentration of 2 μg/10^6^ cells to evaluate T cell cytokine production or with CpG (1 μg/10^6^ cells) to evaluate AID mRNA expression. For H1N1 as well as for the other antigens (data not shown) this is the best stimulating dose generating optimal responses. At the end of stimulation, cells were harvested, supernatants collected for ELISA tests and RNA extracted for quantitative (q)PCR to evaluate AID and GAPDH mRNA expression. Although B cells in the PBMC cultures have been stimulated in the presence of other cell types, primarily T cells and monocytes, our endpoint is to measure a B cell response, as AID is exclusively expressed in B cells [42] and we have previously established that purified B cells give similar results as do PBMC to evaluate effects of aging on the vaccine response [34].

### RNA extraction and quantitative (q)PCR

mRNA was extracted from stimulated PBMC (10^6^ cells), using the μMACS mRNA isolation kit (Miltenyi Biotec) and qPCR performed as described [34,35]. Primers are from Life Technologies.

### Statistical analyses

Non-parametric analyses of the variables were performed by Mann-Whitney test (two-tailed), whereas correlations were performed by Pearson test, using GraphPad Prism 5 software.

## Abbreviations

AID: Activation-induced cytidine deaminase; CSR: Class switch recombination; HAI: Hemagglutination inhibition assay; PBMC: Peripheral blood mononuclear cells.

## Competing interests

No potential conflicts of interest relevant to this article are reported.

## Authors’ contributions

DF designed, performed and supervised the experiments and wrote the paper; AD, MR, NVM and AML performed the experiments; BBB designed and supervised the experiments and wrote the paper. All authors reviewed, edited and approved the manuscript.
